# Straw incorporation and nitrogen reduction effect on the uptake and use efficiency of nitrogen as well as soil CO_2_ emission of relay strip intercropped soybean

**DOI:** 10.3389/fpls.2022.1036170

**Published:** 2022-11-09

**Authors:** Benchuan Zheng, Ping Chen, Qing Du, Huan Yang, Kai Luo, Xiaochun Wang, Feng Yang, Taiwen Yong, Wenyu Yang

**Affiliations:** ^1^ College of Agronomy, Sichuan Agricultural University/Sichuan Engineering Research Center for Crop Strip Intercropping System/Key Laboratory of Crop Ecophysiology and Farming System in Southwest, Ministry of Agriculture, Chengdu, China; ^2^ Crop Research Institute, Sichuan Academy of Agricultural Sciences/Scientific observing and experimental Station of oil crops in the upper Yangtze River, Ministry of Agriculture, Chengdu, China

**Keywords:** straw incorporation, nitrogen uptake, relay strip intercropping, soybean, carbon dioxide emission

## Abstract

Intercropping can increase crop N uptake and reduce carbon emissions. However, the effects of straw incorporation and N reduction on N use and carbon emissions in intercropping are still unclear. We explored the mechanism of N uptake, N use efficiency, and CO_2_ emissions in the wheat-maize-soybean relay strip intercropping system. A two-year field experiment was conducted with two straw managements, i.e., wheat straw incorporation (SI) and straw removal (SR), and four N application levels of soybean, i.e., 60 (N60), 30 (N30), 15 (N15), and 0 kg N ha^-1^ (N0). We assessed soil properties, CO_2_ emissions, and characteristics of roots, nodules, and aboveground N uptake of intercropped soybean. Results showed that geometry mean diameter of aggregate, soil porosity, soil total N, and soil urease activity were notably greater in SI than in SR. N input reduced from N60 to N30 did not significantly affect the soil total N content and urease activity in SI. The root length, root surface area, root volume, root biomass, root bleeding intensity, and inorganic N content of bleeding sap were greater in SI than in SR. In the SI, although the root length and surface area peaked at N60, the root biomass and inorganic N content of bleeding sap were insignificant between N60 and N30. The nodule number, nodule dry weight, nodule nitrogenase activity, and nodule nitrogen fixation potential in SI were notably increased compared with SR. The nodule nitrogen fixation potential in SI notably increased with the decrease of N input at the R3 stage, but it peaked in N30 at the R5 stage. On average, the aboveground N uptake and nitrogen recovery efficiency (RE) was notably higher by 43.7% and 76.8% in SI than in SR. SI+N30 achieved the greatest aboveground N uptake and RE. The CO_2_ emission and accumulated CO_2_ emission were notably greater in SI than in SR, and the accumulated CO_2_ emission of SI was the lowest with N30 input. In conclusion, SI+N30 promoted N uptake and utilization efficiency with reduced CO_2_ emissions during the soybean cropping season. It provides a potential strategy for sustainable agricultural development in intercropping systems.

## Introduction

Food security has become a severe issue with the world’s population and food consumption growth. Modern strengthened agriculture production can meet food security to some degree, while it largely depends on high chemical fertilizer input, e.g., nitrogen (Sinclair and Rufty, 2012). High nitrogen (N) input leads to severe environmental risks, e.g., greenhouse gas emissions, water pollution, and soil acidification (Goldblatt et al., 2009). Sustainable agricultural production management strategies are urgently needed to achieve food security. Intercropping has been recognized as a sustainable agricultural development model globally due to the yield advantages, efficient use of resources, and maintain soil fertility (Nyawade et al., 2019; Du et al., 2020; Fan et al., 2020). The wheat-maize-soybean relay strip intercropping system is a mainly planting pattern in southwestern regions of China. It has the advantages of reducing N fertilizer input, eliminating environmental pollution, and boosting the system yield (Yong et al., 2012; Yong et al., 2015). However, considerable wheat straws were burned or removed, and the overuse of N fertilizer for soybean in the system resulted in resource waste and environmental pollution. Straw incorporation could increase farmland productivity by improving soil properties, e.g., increasing soil organic matter (SOM) and nutrients (Chen et al., 2018; Dong et al., 2018; Ma et al., 2019), enhancing soil aggregates formation (Song et al., 2019; Bu et al., 2020), and optimizing microbial community diversity and composition (Zhao et al., 2016; Cong et al., 2020). Especially, straw incorporation can protect soil from water erosions in areas with heavy rainfall or wind erosions in sandy-sloping farmland (Li et al., 2018). Straw removed from the field can be used for burning or bioenergy due to the unrenewable fossil energy, but reusing crop straw may result in environmental pollution (Zhao et al., 2019). Therefore, straw incorporation provides a potential way to reduce chemical fertilizer input and decrease the risk of environmental pollution (Asten et al., 2005; Ma et al., 2019; Latifmanesh et al., 2020).

The root system is the main organ through which plants use soil nutrients and water. Thus, crop growth and yields affect by proliferating roots in nutrient-enriched regions and increasing soil nutrient uptake (James et al., 2008; Chilundo et al., 2017; Ramamoorthy et al., 2017). To clear the root growth in the soil and the relationship between root, soil structure, and soil nutrients are in favor of increasing grain yield and the efficiency of fertilizer use (Xu et al., 2018; Zheng et al., 2021; Zheng et al., 2022). Agriculture management, e.g., straw incorporation and N application, can improve the structure and function of the root system by regulating soil structure, increasing soil structure stability, and affecting the spatial distribution of soil nutrient**s** (Yu et al., 2017; Xu et al., 2018; Yu et al., 2018). Straw incorporation favors decreasing soil bulk density and increasing soil total porosity, which improves soil ventilation conditions and promotes root penetration in deep soil (Xu et al., 2018). Besides, organic matter in the straw i**s** decomposed and released, which enhances the content of available N, phosphorus, and potassium (Yang et al., 2019). However, the straw decomposition is limited by cereal straws’ high C: N ratio (Shaukat et al., 2011). A reasonable N input balances the C: N ratio and promotes straw decomposition by soil microorganisms (Huang et al., 2019; Li et al., 2021). Moreover, straw incorporation affected the vertical distributions of available nutrients (Yu et al., 2018), which promoted root proliferation. N input can encourage the growth of the root system (Zheng et al., 2022). However, there may be an optimal N rate for root growth, above which root growth may be suppressed. In the ridge film mulching and furrow planting pattern, taproot length, taproot diameter, taproot dry weight, and lateral root mass density of winter oilseed rape peaked at 240 kg N ha^-1^ when N input ranging from 0 to 300 kg N ha^-1^ (Gu et al., 2019). The improved root system favors promoting soil N uptake and increasing N use efficiency (Zheng et al., 2021). Xu et al. (2018) found that straw incorporation coupled with low N input can promote root distribution in deep soil, then increase nitrogen partial factor productivity and agronomic nitrogen use efficiency of winter wheat. However, the effects of straw incorporation and reduced N application on crop root growth in the intercropping system are unclear.

The cereal-legume intercropping system has attracted much attention because of legume symbiotic nitrogen fixation (Zhang et al., 2017; Zheng et al., 2022). The nodulation and N fixation of the legume not only meets its own N needs but also provides additional N sources for the soil. The soil N content affects legumes’ nodulation and nitrogen fixation (Zheng et al., 2022). The biological N fixation (BNF) of legumes is promoted by increasing nodulation when soil N is deficient; in contrast, sufficient N resources will suppress the BNF of legumes (Hu et al., 2016). The interspecific competitive use of N between cereal and legumes strengthens legume’s nodulation and N fixation (Li et al., 2001). Although the legume BNF is suppressed by soil compactness (Siczek A, 2011), straw incorporation can promote BNF by alleviating soil compactness and increasing soil porosity (Xu et al., 2018). Moreover, soybean nodulation and N fixation are promoted by reducing N input in the maize-soybean relay strip intercropping system (Du et al., 2020). However, the effects of straw incorporation and reduced N input on the nodulation and N fixation of soybean in the intercropping system are unclear.

Straw incorporation and N application increased crop production and produced adverse environmental effects, e.g., increased carbon dioxide (CO_2_) emissions (Bhattacharyya et al., 2012; Shao et al., 2014). Then, mitigation of CO_2_ emissions will be an essential task for the sustainable development of agriculture. Chai et al. (2013) indicated that maize intercropped with rape, pea, and wheat can decrease carbon emissions per unit area compared with monoculture maize in arid irrigation areas. Similarly, wheat-maize intercropping can reduce soil carbon emission in contrast to monoculture maize, and zero tillage with straw mulching has the lowest soil respiration rate Hu et al. (2015). Accordingly, the carbon emission is reduced by 12.4% compared to tillage without straw retention. Zhang et al. (2020) observed that reduction N application significantly decreased CO_2_ emissions in contrast to conventional N application under the straw incorporation treatment in Huang-Huai-Hai wheat-maize rotation areas of China. Hence, intercropping and N reduction application provides possible ways to reduce carbon emissions. However, the effects of straw incorporation and reduced N input on soil CO_2_ emissions in intercropping systems are still unclear and need better understood.

Our previous study indicated that maize-soybean relay strip intercropping could increase N uptake by changing spatial root distribution, promoting the BNF of soybean (Zheng et al., 2022). Further, reduced Ninput can enhance BNF by strengthening interspecific competitive use N (Du et al., 2020). However, it is unclear whether or not straw incorporation coupled with reduced N application can improve N uptake and utilization, and the mechanisms of N utilization and environmental effects are still clouded in wheat-maize-soybean relay strip intercropping. We hypothesized that wheat straw incorporation coupled with reduced N application would promote N use in an environment-friendly way by improving soil properties, strengthening soybean root growth and N fixation, and decreasing soil CO_2_ emissions in the wheat-maize-soybean relay strip intercropping system. The objectives of this study were to (1) clarify the influence of straw incorporation coupled with reduced N application on the soil properties, root growth, nodulation, and N uptake and use efficiency of soybean and (2) evaluate the characteristic of CO_2_ emissions in the wheat-maize-soybean relay strip intercropping system.

## Materials and methods

### Site description

The experimental site is located in Renshou County (30°16’N, 104°00’E), Sichuan Province, Southwest China, in the 2018-2019 and 2019-2020 growing seasons. The climate of this region is subtropical monsoon humidity, with an average annual precipitation of 1110.7 mm and a temperature of 17.9°C. The precipitation and temperature during the soybean cropping seasons are shown in **supplementary Figure1**. The soil in this region is anthrosol with a clay loam texture. The soil organic matter, total N, total P, total K, and pH were 7.85 g kg^-1^, 0.61 g kg^-1^, 0.84 g kg^-1^, 22.66 g kg^-1^, and 8.21, respectively. The wheat-maize-soybean relay strip intercropping system was the main planting pattern in this region.

### Experimental design and field management

The experiment site was set up on a fallow field. A two-factor split-plot experimental design was carried out with three replicates. The main factor was straw management with full straw incorporation (SI) and complete straw removal (SR). The sub-factor was N application rates of intercropped soybean, including conventional N employed by local farmers (N60, 60 kg N ha^-1^), reduced N by 50% (N30, 30 kg N ha^-1^), reduced N by 75% (N15, 15 kg N ha^-1^), and zero N (N0). In the SI treatment, all wheat straw was crushed into pieces (0.05 m) and incorporated into the soil by rotary tillage (about 20 cm depth) after wheat harvest every year. In the SR treatment, all wheat straws were removed from the field, and the crops stubble less than 5 cm in height.

In the wheat-maize-soybean relay strip intercropping system, a wide-narrow row planting was adopted (1.6 m and 0.4 m for wide and narrow rows), and a total ratio of wheat-to-maize-to-soybean rows was 4:2:2. Wheat was planted in the wide rows with row spacings of 0.25 m as a first crop. Then, maize was sown in narrow rows with row spacings of 0.4 m. The distance was 0.425 m between wheat and maize rows. After the wheat harvest, two rows of soybean were sown in the wheat strips with row spacings of 0.4 m, which was 0.6 m between maize and soybean (**supplementary Figure 2**). The plot size was 5 m × 6 m. The planting density of wheat, maize, and soybean was 2,000,000 plants ha^-1^, 58,863 plants ha^-1^, and 117,726 plants ha^-1^, respectively. The N, P, and K fertilizers were applied as urea (46% N), superphosphate (12% P_2_O_5_), and potassium chloride (60% K_2_O), respectively. N fertilizer for wheat (150 kg N ha^-1^) and soybean was applied as basal fertilizer, while N for maize was divided into two parts, i.e., 120 kg N ha^-1^ as basal fertilizer and 120 kg N ha^-1^ as topdressing. The P and K fertilizers were applied as base fertilizers for each crop, i.e., 36 kg P_2_O_5_ ha^-1^ and 54 kg K_2_O ha^-1^ for wheat, 120 kg P_2_O_5_ ha^-1^ and 120 kg K_2_O ha^-1^ for maize, and 60 kg P_2_O_5_ ha^-1^ and 52.5 kg K_2_O ha^-1^ for soybean. Wheat fertilizers were broadcast in the planting strips and incorporated into the topsoil (20 cm) by a rotary tiller. Maize fertilizers were strip placed in the middle of two maize planting rows at a distance of 20 cm from maize rows. Soybean fertilizers were hole placed at 10 cm from the soybean. The fertilizers for maize and soybean were hand-placed at a depth of 10 cm. In the 2018-2019 growing season, wheat was sown on November 15, 2018 and harvested on May 14, 2019, maize was sown on April 9, 2019 and harvested on July 27, 2019; soybean was sown on June 8, 2019 and harvested on November 3, 2019. In the 2019-2020 growing season, wheat was sown on November 14, 2019 and harvested on May 8, 2020, maize was sown on April 5, 2020 and harvested on July 29, 2020, soybean was sown on June 9, 2020 and harvested on October 28, 2020. The cultivars of wheat (*Triticum aestivum* L.), maize (*Zea mays L*.), and soybean (*Glycine max* L*. Merr.*) were Zhongkemai-138, Denghai-605, and Nandou-25, respectively.

### Sampling and measurement

Soil samples of soybean were collected from each plot at a depth of 0-20 cm using soil anger (2 cm diameter and 20 cm depth) at the fifth trifoliolate stage (V5, July 17, 2019 and July 21, 2020), the beginning seed stage (R5, August 29, 2019 and September 8, 2020), and the full-maturity stage of soybean (R8, November 3, 2019 and October 28, 2020). Three individual samples were collected per plot, then thoroughly mixed and sieved through a 2 mm mesh to remove plant tissues, roots, and rocks. The fresh soil samples were stored at -80°C for the soil urease activity analysis (Zheng et al., 2022). The soil samples were air-dried to investigate the total nitrogen (TN) content (Liu et al., 2021).

Soil samples for soil aggregates and bulk density (BD) assessment were collected after the soybean harvest. Soil clods were collected at 0-20 cm soil depth. Within each plot, five individual soil samples were collected. The fresh soil was gently stripped into 10-12 mm clods along the natural planes of weakness, then air-dried for soil aggregation analysis. Soil aggregate separation was performed according to Guo et al. (2020). Two undisturbed soil cores from each plot at 0-10 cm and 10-20 cm depths with a volume of 100 cm^3^ were collected for soil BD measurement. Soil samples were oven-dried at 105°C for 24 h, long enough to reach constant weight for weighting and BD calculation (Xu et al., 2018).

Plant samples of soybean were collected at the fifth trifoliolate stage (V5, July 17, 2019 and July 21, 2020), the beginning flowering stage (R1, July 31, 2019 and August 5, 2020), the beginning pod stage (R3, August 15, 2019 and August 24, 2020), the beginning seed stage (R5, August 29, 2019 and September 8, 2020), and the full-maturity stage (R8, November 3, 2019 and October 28, 2020). In each treatment, six soybean plants were cut from the first internode, and the aboveground samples were dried at 85°C to constant weight and weighting, then ground and passed through a 60-mesh sieve (0.25 mm) for plant N content measurement (Zheng et al., 2021). The underground roots use a traditional excavation method to obtain 0.20 m × 0.20 m × 0.20 m soil clods (Zheng et al., 2021). Soybean roots were scanned at a 300 dpi resolution (Epsom expression 10000 XL (Japanese) Co., Ltd). The scanned root images were analyzed using Win-RHIZO™ software (Régent Instruments Inc., Canada). Then the root samples were dried at 85 °C to a constant weight.

Root bleeding sap samples were collected at the beginning-pod stage (R3) and the beginning-seed stage (R5) of soybean. The collection method was modified from Zheng et al. (2021). Namely, soybeans were cut 10 cm above the soil surface. Then, skimmed cotton was put into a self-sealing bag. The weighed self-sealing bag was placed on the soybean stalk and fixed with a rubber band. The bleeding sap in the skimmed cotton was collected and weighed after 12 h (6:00 pm - 6:00 am). The bleeding sap intensity was calculated as bleeding sap weight per plant per 12h (g plant^−1^ 12h^−1^). The ammonium-N and nitrate-N content of bleeding sap was measured using a Cleverchem Anna Random Access Analyzer (DeChem-Tech.GmbH-Hamburg, Germany).

Nodule samples were collected at the V5, R1, R3, and R5 stages of soybean. In each plot, six representative plants were dug out to investigate the nodules’ number and weight according to the early study (Yong et al., 2018). In 2020, the nodule nitrogenase activity was measured by acetylene reduction assay (C_2_H_2_) at the R3 and R5 stages of soybean (Siczek A, 2011). The nodule nitrogen fixation potential was evaluated according to Yong et al. (2018).

To explore the effects of straw incorporation and N application rates on the environment. The soil CO_2_ emission rate was measured by a soil carbon flux meter (Brooke soilbox-343 portable soil respiration system, Germany) at the V5, R1, R3, R5, and R8 stages of soybean in 2020. The measuring site was set at the interspecific rows between maize and soybean, with a distance of 20 cm from the soybean row.

### Calculations and statistics

The soils’ total porosity was evaluated according to soil bulk density and calculated as follows (Yang et al., 2021):


(1) $$P=100\times \left(1-\frac{B}{S}\right)$$

Where P is total soil porosity (%), *B* is soil bulk density (g cm^-3^), and *S* is soil density (2.65 g cm^-3^).

The soils’ macroaggregate mechanical stability was evaluated using Mean weight diameter (MWD) and Geometry mean diameter (GMD), and calculated as follows (Kihara et al., 2012):


(2) $$\text{MWD}\;(\text{mm})=\frac{\sum_{i=1}^{n}X_{i}W_{i}}{\sum_{i=1}^{n}W_{i}}$$

(3) $$GMD\;(mm)=Exp\left[\frac{\sum_{i=1}^{n}W_{i}\ln x_{i}}{\sum_{i=1}^{n}W_{i}}\right]$$

Where X_i_ is the average diameter of grade i aggregates (mm), and W_i_ is the mass ratio of grade i aggregates (%).

The N use efficiency (NUE) of different treatments was evaluated using recovery efficiency (RE), and calculated according to Zheng et al. (2021).


(4) $$RE\%=\frac{U_{N}-U_{O}}{F_{N}}$$

Where *U_N_* is the aboveground N uptake with N, *U_0_* is the aboveground N uptake without N, and *F_N_* is the N application rates, respectively.

The cumulative CO_2_ emission (CE) was calculated according to Hu et al. (2015)


(5) $$\text{CE(kg}\;{\text{ha}}^{-1})=\sum \left[\frac{F_{n+1}+F_{n}}{2}\times (T_{n+1}-T_{n})\right]\times 60\times 24\times \frac{1}{100}\times \frac{B}{A}$$

Where the F_n_ and F_n+1_ were the CO_2_ emission rate of the n and n+1 sampling times (mg m^-2^ min^-1^), T_n_ and T_n+1_ were sampling times of n and n+1(d), 60 and 24 were the conversion of min^-1^ to d^-1^, B is the relative molecular mass ratio (C: CO_2_), and A is warming potential coefficient (1).

Statistical significance was performed with the two-way analysis of variance (ANOVA), and the multiple comparisons were conducted with the least significant difference test (LSD, α=0.05) to determine the differences between individual treatment means. The analyses were performed with SPSS v.22 (IBM Corp., Armon, NY Inc, USA) and Microsoft Excel. SigmaPlot v.14.0 (Systat Software Inc. USA) and Origin 2017 (OriginLab Corporation, USA) were used to draw the figures.

## Results

### Soil physicochemical properties

**Figure 1:**
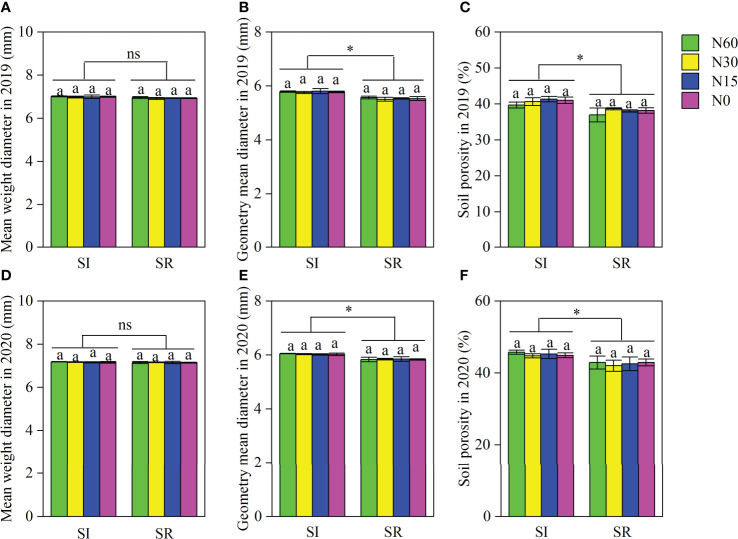
Soil physical properties of intercropped soybean at full maturity stage. The same lower-case letters indicate insignificant differences under different N application rates (*LSD*, *P*>0.05). Data were shown as mean ± S.D. (n=3). The asterisk “*” and “ns” indicate significant differences (*P*<0.05) and insignificant differences (*P*>0.05) between straw incorporation and straw removal, respectively. SI, straw incorporation; SR, straw removal; N60, convention N (60 kg N ha^-1^); N30, reduced N by 50% (30 kg N ha^-1^); N15, reduced N by 75% (15 kg N ha^-1^); N0, zero N (0 kg N ha^-1^). **(A)**, mean weight diameter in 2019; **(B)** geometry mean diameter in 2019; **(C)** soil porosity in 2019; **(D)**, mean weight diameter in 2020; **(E)** geometry mean diameter in 2020; **(F)** soil porosity in 2020.

As shown in **Figure 1**, the MWD, GMD, and soil porosity were insignificant among N application rates, but the GMD and soil porosity were notably greater in SI than in SR (**Figures 1B, C-E,F**). Straw incorporation and N application significantly influenced soil chemical properties (**Table 1**). The soil TN content and urease activity were markedly higher in SI than in SR under different N application rates at the various growth stages of soybean. Independent with N application rates, the soil TN content was significantly greater by 8.9%~16.7% in SI than in SR at the R8 stage of soybean. There were no significant differences in soil TN content and urease activity between N60 and N30 under the SI treatment at the different growth stages of soybean. But, those were significantly lower in N15 and N0 than in N30 and N60. Compared with N60, the soil TN and urease activity significantly decreased with the decrease of N inputs under the SR treatment at the different growth stages of soybean.

**Table 1:** Soil chemical properties of intercropped soybean at different growth stages.

Years	N application rates	Total nitrogen content (TN, g kg^-1^)													Soil urease activity (μg d^-1^ g^-1^)							
		V5-stage		R5-stage				R8-stage					V5-stage					R5-stage			
		SI	SR	SI		SR		SI			SR		SI			SR		SI		SR	
2019	N60	0.83 ± 0.01 a	0.77 ± 0.01 a	0.80 ± 0.03 a		0.74 ± 0.01 a		0.83 ± 0.01 a			0.75 ± 0.01 a		344.7 ± 4.8 a			317.6 ± 2.6 a		274.6 ± 4.2 a		251.1 ± 3.7 a	
	N30	0.82 ± 0.01 a	0.72 ± 0.01 b	0.77 ± 0.04 a		0.68 ± 0.00 b		0.82 ± 0.01a			0.70 ± 0.01b		343.6 ± 6.3 a			295.0 ± 7.2 b		263.2 ± 9.3 a		223.2 ± 4.9 b	
	N15	0.70 ± 0.01 b	0.66 ± 0.02 c	0.70 ± 0.01 b		0.63 ± 0.01 c		0.76 ± 0.00 b			0.65 ± 0.01c		292.4 ± .6 b			274.9 ± 6.4 c		231.9 ± 4.3 b		199.0 ± 10.4 c	
	N0	0.67 ± 0.02 c	0.64 ± 0.04 c	0.64 ± 0.00 c		0.57 ± 0.00 d		0.70 ± 0.01 c			0.60 ± 0.01 d		287.4 ± 0.9 b			249.8 ± 1.3 d		192.2 ± 7.6 c		178.0 ± 2.6 d	
2020	N60	0.86 ± 0.02 a	0.79 ± 0.03 a	0.78 ± 0.00 a		0.73 ± 0.01 a		0.82 ± 0.00 a			0.75 ± 0.01 a		354.0 ± 5.9 a			325.0 ± 7.4 a		418.9 ± 7.1 a		386.7 ± 5.3 a	
	N30	0.84 ± 0.03 a	0.74 ± 0.02 b	0.78 ± 0.01 a		0.66 ± 0.01 b		0.80 ± 0.02 a			0.70 ± 0.01 b		346.5 ± 4.0 a			291.3 ± 10.1 b		415.7 ± 4.6 a		355.4 ± 1.9 b	
	N15	0.76 ± 0.00 b	0.70 ± 0.02 bc	0.68 ± 0.00 b		0.63 ± 0.03 c		0.72 ± 0.00 b			0.64 ± 0.00 c		296.6 ± 3.0 b			280.9 ± 4.9 b		382.6 ± 6.9 b		346.9 ± 11.1 bc	
	N0	0.74 ± 0.01 b	0.68 ± 0.01 c	0.63 ± 0.01 c		0.57 ± 0.01 d		0.66 ± 0.01 c			0.60 ± 0.01 d		297.1 ± 15.4 b			280.4 ± 6.5 b		367.2 ± 4.7 c		329.6 ± 17.4 c	
ANOVA (*F*-value)																						
Year (Y)		51.2^*^			3.5^ns^					25.8^*^					19.2^*^					4554.7^*^		
Straw management (S)		154.0^*^			217.4^*^					1047.3^*^					264.4^*^					245.7^*^		
N application rate (N)		134.7^*^			207.2^*^					582.3^*^					199.6^*^					170.6^*^		
Y×S		2.1^ns^			0.1^ns^					15.8^*^					0.9^ns^					9.8^*^		
Y×N		2.3^ns^			0.1^ns^					1.9^ns^					5.3^*^					5.7^*^		
S×N		6.5^*^			4.4^*^					10.7^*^					15.4^*^					6.2^*^		
Y×S×N		0.7^ns^			0.8^ns^					1.6^ns^					2.5^ns^					1.2^ns^		

Different lower-case letters indicate significant differences under different N application rates (LSD, P< 0.05). Data were shown as mean ± S.D. (n=3); The asterisk “*” and “ns” indicate significant difference (P< 0.05) and insignificant difference (P >0.05), respectively; SI, straw incorporation; SR, straw removal; N60, convention N (60 kg N ha^-1^); N30, reduced N by 50% (30 kg N ha^-1^); N15, reduced N by 75% (15 kg N ha^-1^); N0, zero N (0 kg N ha^-1^); V5, the fifth trifoliolate stage of soybean; R5, the beginning seed stage of soybean; R8, the full-maturity stage of soybean.

### Root configuration and root biomass

**Figure 2:**
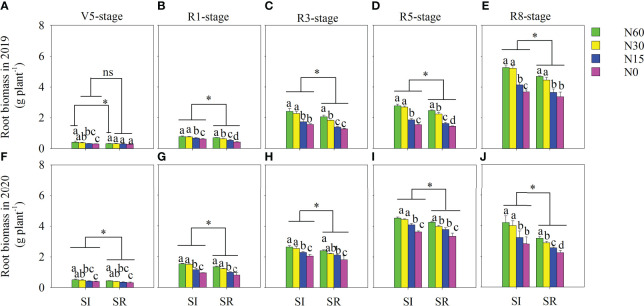
Effects of straw incorporation and nitrogen application on root biomass of intercropped soybean at the different growth stages. Different lower-case letters indicate significant differences under different N application rates (*LSD*, *P*< 0.05). Data were shown as mean ± S.D. (n=3). The asterisk “*” and “ns” indicate significant differences (*P*< 0.05) and insignificant differences (*P* >0.05) between straw incorporation and straw removal, respectively. SI, straw incorporation; SR, straw removal; N60, convention N (60 kg N ha^-1^); N30, reduced N by 50% (30 kg N ha^-1^); N15, reduced N by 75% (15 kg N ha^-1^); N0, zero N (0 kg N ha^-1^); V5, the fifth trifoliolate stage of soybean; R1, the beginning flowering stage of soybean; R3, the beginning pod stage of soybean; R5, the beginning seed stage of soybean; R8, the full-maturity stage of soybean. **(A)**, root biomass at the V5 stage in 2019; **(B)**, root biomass at the R1 stage in 2019; **(C)**, root biomass at the R3 stage in 2019; **(D)**, root biomass at the R5 stage in 2019; **(E)**, root biomass at the R8 stage in 2019; **(F)**, root biomass at the V5 stage in 2020; **(G)**, root biomass at the R1 stage in 2020; **(H)**, root biomass at the R3 stage in 2020; **(I)**, root biomass at the R5 stage in 2020; **(J)**, root biomass at the R8 stage in 2020.

The root length, surface area, and volume were significantly affected by straw incorporation and N application at the different growth stages of soybean (**Tables 2**–**4**). Independent with N application rates, those were significantly higher in SI than in SR at different growth stages of soybean (**Tables 2**–**4**). Compared with N60, the root length and surface area were increased in N30 but significantly decreased in N15 and N0 under the SI treatment at the different growth stages of soybean (**Tables 2**–**3**). However, the root length and surface area were considerably reduced in N30, N15, and N0 in contrast to N60 under the SR treatment (**Tables 2**–**3**). Compared with SR, the root biomass was notably increased in SI (**Figure 2**). On average, the root biomass of SI significantly increased by 21.6%, 27.4%, 19.9%, and 18.3% compared with SR under the N60, N30, N15, and N0 treatments at the R8 stage (**Figures 2E-J**). With the decrease of N, although root biomass was insignificant between N60 and N30 in SI, it was significantly decreased in N15 compared with N30. Similarly, root biomass was notably reduced with the decrease of N in SR treatment.

**Table 2:** Effects of straw incorporation and nitrogen application on root length of intercropped soybean at different growth stages (cm plant^-1^).

Years	N application rates	V5-stage		R1-stage				R3-stage					R5-stage				
		SI	SR	SI		SR		SI			SR		SI			SR	
2019	N60	462.4 ± 29.5a	388.6 ± 20.8a	930.1 ± 5.3b		862.1 ± 8.5a		1173.1 ± 8.2b			1013.0 ± 7.3a		1576.8 ± 89.6a			1325.9 ± 92.1a	
	N30	468.5 ± 31.7a	387.9 ± 23.0a	957.1 ± 10.8a		729.9 ± 123.4b		1232.7 ± 33.1a			949.3 ± 26.1b		1591.9 ± 2.7a			1122.3 ± 69.4b	
	N15	385.1 ± 50.8b	280.0 ± 20.5b	847.3 ± 33.9c		719.8 ± 51.2b		1060.8 ± 56.8c			843.3 ± 22.1c		1335.1 ± 41.0b			1066.3 ± 26.3bc	
	N0	355.0 ± 61.4b	277.3 ± 25.8b	836.8 ± 30.2c		705.1 ± 15.8b		1002.2 ± 53.3c			583.4 ± 51.7d		1074.0 ± 89.1c			994.6 ± 9.0c	
2020	N60	646.3 ± 32.2ab	563.7 ± 38.1a	1363.7 ± 5.7b		1245.0 ± 43.8a		2453.3 ± 13.6a			2244.6 ± 9.6a		1349.8 ± 71.7a			1199.5 ± 56.5a	
	N30	660.6 ± 60.3a	543.2 ± 22.5a	1536.5 ± 51.0a		1143.2 ± 18.2b		2588.4 ± 97.6a			2105.1 ± 4.9b		1413.6 ± 70.7a			1116.8 ± 19.6ab	
	N15	561.4 ± 36.4b	411.9 ± 17.1b	1113.7 ± 45.5c		923.2 ± 98.6bc		1953.1 ± 74.2b			1743.2 ± 8.5c		1214.1 ± 1.8b			1014.8 ± 99.4bc	
	N0	406.8 ± 71.0c	318.2 ± 30.5 b	942.5 ± 19.4d		838.2 ± 7.5c		1851.4 ± 80.1b			1680.0 ± 16.4c		1108.5 ± 4.6b			913.5 ± 26.4c	
ANOVA (*F*-value)																	
Year (Y)		96.6^*^			220.4^*^					2141.9^*^					27.6^*^		
Straw management (S)		47.4^*^			64.4^*^					129.4^*^					175.4^*^		
N application rate (N)		37.4^*^			41.1^*^					90.4^*^					73.3^*^		
Y×S		0.8^ns^			2.2^ns^					0.0^ns^					2.5^ns^		
Y×N		4.9 ^*^			16.0^*^					15.8^*^					3.1^*^		
S×N		0.6^ns^			5.3^*^					3.6^*^					8.4^*^		
Y×S×N		0.1^ns^			0.9^ns^					3.9^*^					2.9^ns^		

Different lower-case letters indicate significant differences under different N application rates (LSD, P< 0.05); Data were shown as mean ± S.D. (n=3); The asterisk “*” and “ns” indicate significant difference (P< 0.05) and insignificant difference (P >0.05), respectively; SI, straw incorporation; SR, straw removal; N60, convention N (60 kg N ha^-1^); N30, reduced N by 50% (30 kg N ha^-1^); N15, reduced N by 75% (15 kg N ha^-1^); N0, zero N (0 kg N ha^-1^); V5, the fifth trifoliolate stage of soybean; R1, the beginning flowering stage of soybean; R3, the beginning pod stage of soybean; R5, the beginning seed stage of soybean.

**Table 3:** Effects of straw incorporation and nitrogen application on root surface area of intercropped soybean at different growth stages (cm^2^ plant^-1^).

Years	N application rates	V5-stage	R1-stage			R3-stage			R5-stage	
		SI	SR	SI	SR		SI	SR	SI	SR
2019	N60	105.0 ± 4.1a	91.9 ± 4.9a	258.6 ± 34.2a	216.3 ± 10.3a		398.3 ± 19.3b	361.5 ± 11.1a	372.7 ± 40.1ab	318.0 ± 21.4a
	N30	108.9 ± 5.9a	94.9 ± 3.2a	270.4 ± 23.0a	187.6 ± 12.4b		460.1 ± 11.8a	305.6 ± 17.4ab	384.3 ± 55.3a	281.0 ± 28.6ab
	N15	93.5 ± 5.6b	73.1 ± 7.2b	202.1 ± 9.3b	171.7 ± 3.8bc		324.2 ± 36.8c	292.7 ± 39.4ab	302.7 ± 11.4bc	261.5 ± 15.1b
	N0	84.2 ± 1.3c	72.8 ± 1.2b	191.1 ± 7.8b	162.7 ± 12.4c		272.6 ± 77.1c	240.9 ± 86.1b	274.8 ± 27.6c	257.1 ± 28.0b
2020	N60	113.2 ± 12.7a	92.8 ± 2.5a	359.1 ± 28.7a	236.8 ± 14.3a		536.8 ± 14.2a	494.5 ± 4.7a	430.4 ± 4.9a	400.9 ± 1.2a
	N30	117.4 ± 7.9a	82.4 ± 7.6ab	388.1 ± 2.2a	211.0 ± 3.8b		554.1 ± 26.4a	444.8 ± 49.8b	436.9 ± 16.6a	382.1 ± 4.5b
	N15	90.2 ± 5.8b	66.2 ± 17.2bc	210.7 ± 5.9b	189.0 ± 11.6c		403.2 ± 39.8b	366.2 ± 30.4c	366.8 ± 27.4b	331.1 ± 20.3c
	N0	71.3 ± 14.2b	61.5 ± 6.2c	187.1 ± 11.9b	144.7 ± 21.8d		392.4 ± 60.2b	326.8 ± 8.0c	303.1 ± 7.3c	276.2 ± 28.3d
ANOVA (*F*-value)										
Year (Y)		2.5^ns^		51.7^*^			83.9^*^		66.6^*^	
Straw management (S)		63.8^*^		219.2^*^			34.1^*^		39.0^*^	
N application rate (N)		38.6^*^		112.9^*^			35.2^*^		44.1^*^	
Y×S		2.7^ns^		23.6^*^			0.1^ns^		1.4^ns^	
Y×N		2.3^ns^		17.6^*^			1.1^ns^		2.8^ns^	
S×N		1.8^ns^		26.8^*^			5.2^*^		2.7^ns^	
Y×S×N		1.1^ns^		7.3^*^			0.2^ns^		0.7^ns^	

Different lower-case letters indicate significant differences under different N application rates (LSD, P< 0.05); Data were shown as mean ± S.D. (n=3); The asterisk “*” and “ns” indicate significant difference (P< 0.05) and insignificant difference (P >0.05), respectively; SI, straw incorporation; SR, straw removal; N60, convention N (60 kg N ha^-1^); N30, reduced N by 50% (30 kg N ha^-1^); N15, reduced N by 75% (15 kg N ha^-1^); N0, zero N (0 kg N ha^-1^); V5, the fifth trifoliolate stage of soybean; R1, the beginning flowering stage of soybean; R3, the beginning pod stage of soybean; R5, the beginning seed stage of soybean.

**Table 4:** Effects of straw incorporation and nitrogen application on root volume of intercropped soybean at different growth stages (cm^3^ plant^-1^).

Years	N application rates	V5-stage		R1-stage			R3-stage				R5-stage			
		SI	SR	SI	SR		SI		SR		SI		SR	
2019	N60	7.2 ± 1.4a	5.6 ± 1.2a	15.2 ± 1.7a	12.2 ± 0.3a		32.6 ± 0.5a		26.8 ± 0.5a		34.0 ± 1.2a		24.9 ± 0.9a	
	N30	6.7 ± 0.1a	5.5 ± 0.4a	14.4 ± 0.3a	9.8 ± 0.5b		30.1 ± 1.0a		23.6 ± 0.7b		30.4 ± 3.8a		18.8 ± 2.7b	
	N15	5.7 ± 0.1b	4.2 ± 0.1b	11.0 ± 1.3b	9.2 ± 1.8b		24.1 ± 4.3b		18.1 ± 1.0c		25.8 ± 1.3b		15.9 ± 1.8b	
	N0	5.6 ± 0.2b	4.0 ± 0.2b	10.10.2 ± b	8.2 ± 0.4b		18.4 ± 1.1c		17.5 ± 2.3c		19.2 ± 0.2c		15.2 ± 2.8b	
2020	N60	4.2 ± 0.6a	3.3 ± 0.2a	14.7 ± 4.2a	11.4 ± 0.2a		31.7 ± 0.4a		28.0 ± 0.7a		32.2 ± 0.6a		27.2 ± 1.4a	
	N30	4.0 ± 0.6a	2.6 ± 0.2b	14.2 ± 2.1a	9.3 ± 0.8b		30.3 ± 0.3a		25.4 ± 0.6b		26.8 ± 1.2b		23.3 ± 0.4b	
	N15	2.4 ± 0.1b	2.1 ± 0.2c	10.6 ± 1.9b	8.5 ± 0.1bc		23.6 ± 1.7b		22.2 ± 1.2c		22.3 ± 0.1c		20.0 ± 3.2bc	
	N0	2.3 ± 0.4b	2.0 ± 0.3c	10.2 ± 0.3b	7.0 ± 1.8c		21.2 ± 0.3c		17.9 ± 4.3c		20.1 ± 2.4c		18.5 ± 1.1c	
ANOVA (*F*-value)														
Year (Y)		310.7^*^		1.6^ns^				4.9^*^				2.1^ns^		
Straw management (S)		52.3^*^		49.5^*^				61.0^*^				113.4^*^		
N application rate (N)		26.2^*^		21.5^*^				92.4^*^				79.5^*^		
Y×S		5.5^*^		0.4^ns^				2.0^ns^				25.6^*^		
Y×N		0.0^ns^		0.0^ns^				0.5^ns^				0.6^ns^		
S×N		0.6^ns^		2.0^ns^				2.3^ns^				3.8^*^		
Y×S×N		1.2^ns^		0.1^ns^				1.8^ns^				1.5^ns^		

Different lower-case letters indicate significant differences under different N application rates (LSD, P< 0.05); Data were shown as mean ± S.D. (n=3); The asterisk “*” and “ns” indicate significant difference (P< 0.05) and insignificant difference (P >0.05), respectively; SI, straw incorporation; SR, straw removal; N60, convention N (60 kg N ha^-1^); N30, reduced N by 50% (30 kg N ha^-1^); N15, reduced N by 75% (15 kg N ha^-1^); N0, zero N (0 kg N ha^-1^); V5, the fifth trifoliolate stage of soybean; R1, the beginning flowering stage of soybean; R3, the beginning pod stage of soybean; R5, the beginning seed stage of soybean.

### Root bleeding intensity and inorganic nitrogen content of sap

**Figure 3:**
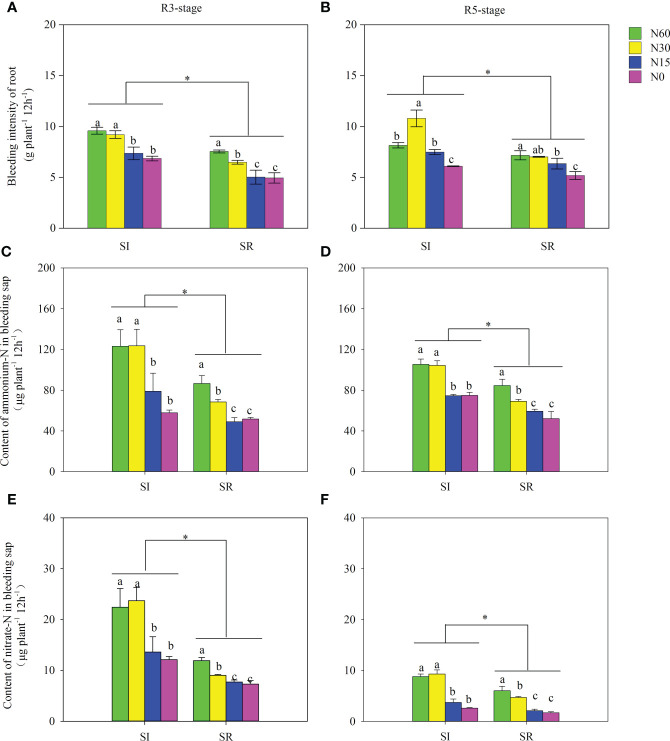
Effects of straw incorporation and nitrogen application on root bleeding intensity and N content at different growth stages. Different lower-case letters indicate significant differences under different N application rates (*LSD*, *P* < 0.05). Data were shown as mean±S.D. (n=3). The asterisk "*" indicate significant differences (*P* < 0.05) between straw incorporation and straw removal, respectively. SI, straw incorporation; SR, straw removal; N60, convention N (60 kg N ha-1); N30, reduced N by 50% (30 kg N ha-1); N15, reduced N by 75% (15 kg N ha-1); N0, zero N (0 kg N ha-1); V5, the fifth trifoliolate stage of soybean; R3, the beginning pod stage of soybean; R5, the beginning seed stage of soybean. **(A)**, bleeding intensity of soybean root at the R3 stage; **(B)**, bleeding intensity of soybean root at the R5 stage; **(C)**, content of ammonium-N in bleeding sap at the R3 stage; **(D)**, content of ammonium-N in bleeding sap at the R5 stage; **(E)**, content of nitrate-N in bleeding sap at the R3 stage; **(F)**, content of nitrate -N in bleeding sap at the R5 stage.

Straw incorporation and N application significantly affected the root bleeding intensity and inorganic N content of bleeding sap (**Figure 3**). Compared with SR, the averaged root bleeding intensity in SI was significantly increased by 38.5% at the R3 stage and by 25.8% at the R5 stage, respectively (**Figures 3A, B**). Compared with N60, no significant differences in root bleeding intensity in N30 were observed under the SI treatment at the R3 stage. However, it significantly decreased in N15 and N0. With the decrease of N, the root bleeding intensity of SR was notably decreased (**Figure 3A**); in contrast, the highest root bleeding intensity of SI was observed in N30 at the R5 stage (**Figure 3B**). The averaged ammonium-N and nitrate-N contents of root bleeding sap were significantly greater in SI than in SR, by 49.0% and 86.4%, respectively, at the R3 stage and by 36.5% and 67.0% at the R5 stage (**Figures 3C-F**). The ammonium-N and nitrate-N contents of bleeding sap in SI showed no significant differences between N60 and N30 (**Figures 3C-F**). In contrast, those were significantly higher in N60 than in N30 under the SR treatment (**Figures 3C-F**).

### Nodule Number and Nodule Dry Weight

**Figure 4:**
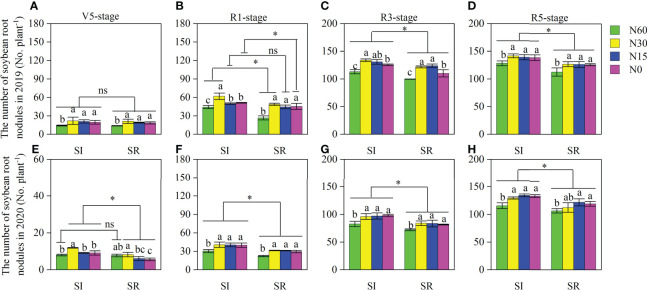
Effects of straw incorporation and N application on root nodule number per plant of intercropped soybean at different growth stages. Different lower-case letters indicate significant differences under different N application rates (*LSD*, *P*< 0.05). Data were shown as mean ± S.D. (n=3). The asterisk “*” and “ns” indicate significant differences (*P*< 0.05) and insignificant differences (*P* >0.05) between straw incorporation and straw removal, respectively. SI, straw incorporation; SR, straw removal; N60, convention N (60 kg N ha^-1^); N30, reduced N by 50% (30 kg N ha^-1^); N15, reduced N by 75% (15 kg N ha^-1^); N0, zero N (0 kg N ha^-1^); V5, the fifth trifoliolate stage of soybean; R1, the beginning flowering stage of soybean; R3, the beginning pod stage of soybean; R5, the beginning seed stage of soybean. **(A)**, the number of soybean root nodules at the V5 stage in 2019; **(B)**, the number of soybean root nodules at the R1 stage in 2019; **(C)**, the number of soybean root nodules at the R3 stage in 2019; **(D)**, the number of soybean root nodules at the R5 stage in 2019; **(E)**, the number of soybean root nodules at the V5 stage in 2020; **(F)**, the number of soybean root nodules at the R1 stage in 2020; **(G)**, the number of soybean root nodules at the R3 stage in 2020; **(H)**, the number of soybean root nodules at the R5 stage in 2020.

**Figure 5:**
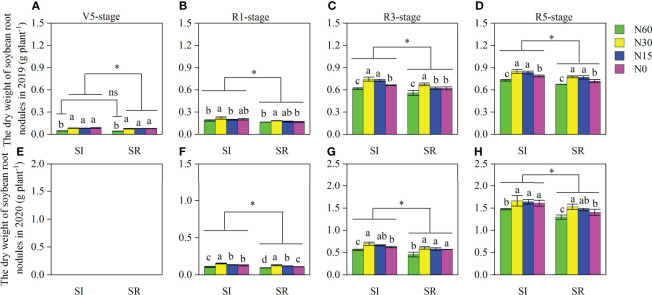
Effects of straw incorporation and N application on root nodule dry weight of intercropped soybean at different growth stages. Due to the small number of nodules per plant and small nodule diameter at the V5 stage in 2020, the nodule weight was not measured. Different lower-case letters indicate significant differences under different N application rates (LSD, *P*< 0.05). The asterisk “*” and “ns” indicate significant differences (*P*< 0.05) and insignificant differences (*P* >0.05) between straw incorporation and straw removal, respectively. Data were shown as mean ± S.D. (n=3). SI, straw incorporation; SR, straw removal; N60, convention N (60 kg N ha^-1^); N30, reduced N by 50% (30 kg N ha^-1^); N15, reduced N by 75% (15 kg N ha^-1^); N0, zero N (0 kg N ha^-1^); V5, the fifth trifoliolate stage of soybean; R1, the beginning flowering stage of soybean; R3, the beginning pod stage of soybean; R5, the beginning seed stage of soybean. **(A)**, the dry weight of soybean root nodules at the V5 stage in 2019; **(B)**, the dry weight of soybean root nodules at the R1 stage in 2019; **(C)**, the dry weight of soybean root nodules at the R3 stage in 2019; **(D)**, the dry weight of soybean root nodules at the R5 stage in 2019; **(E)**, the dry weight of soybean root nodules at the V5 stage in 2020; **(F)**, the dry weight of soybean root nodules at the R1 stage in 2020; **(G)**, the dry weight of soybean root nodules at the R3 stage in 2020; **(H)**, the dry weight of soybean root nodules at the R5 stage in 2020.

Straw incorporation and N application significantly affected the number and dry weight of root nodules at different growth stages of soybean (**Figures 4** and **5**). Those were markedly greater in SI than in SR under different N application rates at most of the growth stages, except for the V5 stage in 2019. Compared with N60, the number and dry weight of nodules were significantly increased in N30, N15, and N0 under both SI and SR treatments at different growth stages of soybean. With the decrease of N, nodules’ number and dry weight were decreased. The number and dry weight of nodules were greater at the R5 stage than at other stages. On average, the nodules number and dry weight of SI were significantly greater by respectively 11.4%, 12.5%, 12.1%, and 14.5%, 12.7%, 8.8% in N30, N15, and N0 than in N60 at the R5 stage (**Figures 4D-H**, **5D-H**). The nodules number and dry weight of SR were notably increased by 9.3%, 13.3%, and 1.9%, respectively, and by 16.2%, 13.3%, 7.3% (**Figures 4D-H**, **5D-H**). Compared with SR, the number and dry weight of nodules were significantly increased by 10.8%, 13.1%, 9.9%, and 11.0% in SI, respectively, under the N60, N30, N15, and N0 treatments at the R5 stage of soybean (**Figures 4D-H**, **5D-H**).

### Nodule nitrogenase activity and nodule nitrogen fixation potential

**Figure 6:**
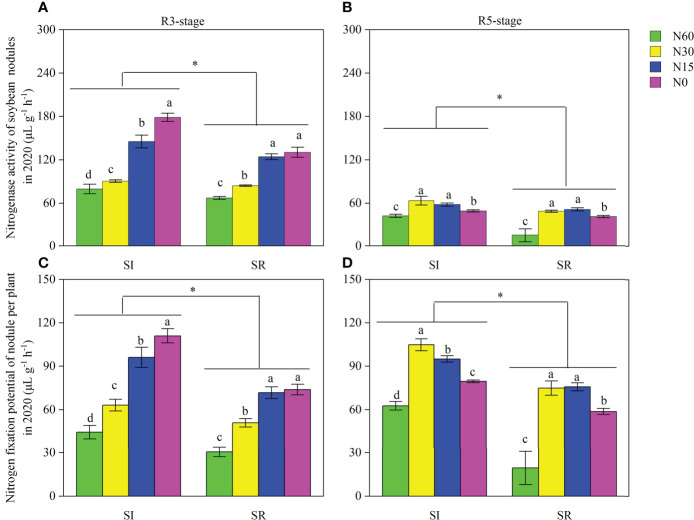
of straw incorporation and N application on nodule nitrogenase activity and nitrogenase fixation potential of intercropped soybean at different growth stages (in 2020). Different lower-case letters indicate significant differences under different N application rates (LSD, *P*< 0.05). The asterisk “*” indicate significant differences (*P*< 0.05) between straw incorporation and straw removal. Data were shown as mean ± S.D. (n=3). SI, straw incorporation; SR, straw removal; N60, convention N (60 kg N ha^-1^); N30, reduced N by 50% (30 kg N ha^-1^); N15, reduced N by 75% (15 kg N ha^-1^); N0, zero N (0 kg N ha^-1^); R3, the beginning pod stage of soybean; R5, the beginning seed stage of soybean. **(A)**, nitrogenase activity of soybean nodules at the R3 stage in 2020; **(B)**, nitrogenase activity of soybean nodules at the R5 stage in 2020; **(C)**, nitrogen fixation potential of soybean nodule per plant at the R3 stage in 2020; **(D)**, nitrogen fixation potential of soybean nodule per plant at the R5 stage in 2020.

Nodules’ nitrogenase activity and nitrogen fixation potential were notably affected by straw incorporation and N input at the R3 and R5 stages (**Figure 6**). Compared with SR, the averaged nitrogenase activity and nitrogen fixation potential of nodules were significantly higher in SI by 7.6%~37.0% and 24.0%~50.3% at the R3 stage (**Figures 6A-C**) and by 13.0%~181.9% and 25.3%~223.2% at the R5 stage (**Figure 6B-D**), respectively. The nitrogenase activity and nitrogen fixation potential of nodules were markedly increased in N30, N15, and N0 compared to N60 under both SI and SR treatments at the R3 and R5 stages. Those were significantly increased with the decrease of N input at the R3 stage and significantly decreased at the R5 stage (**Figure 6**).

### Nitrogen uptake and NUE

**Figure 7:**
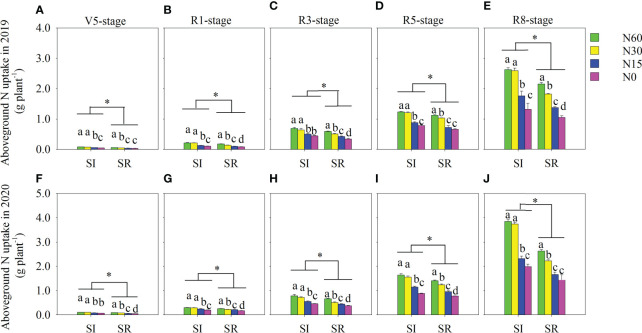
Effects of straw incorporation and nitrogen application on aboveground N-uptake of intercropped soybean at different growth stages. Different lower-case letters indicate significant differences under different N application rates (*LSD*, *P*< 0.05). Data were shown as mean ± S.D. (n=3). The asterisk “*” indicates significant differences (*P*< 0.05) between straw incorporation and straw remove. SI, straw incorporation; SR, straw removal; N60, convention N (60 kg N ha^-1^); N30, reduced N by 50% (30 kg N ha^-1^); N15, reduced N by 75% (15 kg N ha^-1^); N0, zero N (0 kg N ha^-1^); V5, the fifth trifoliolate stage of soybean; R1, the beginning flowering stage of soybean; R3, the beginning pod stage of soybean; R5, the beginning seed stage of soybean; R8, the full-maturity stage of soybean. **(A)**, aboveground N uptake at the V5 stage in 2019; **(B)**, aboveground N uptake at the R1 stage in 2019; **(C)**, aboveground N uptake at the R3 stage in 2019; **(D)**, aboveground N uptake at the R5 stage in 2019; **(E)**, aboveground N uptake at the R8 stage in 2019; **(F)**, aboveground N uptake at the V5 stage in 2020; **(G)**, aboveground N uptake at the R1 stage in 2020; **(H)**, aboveground N uptake at the R3 stage in 2020; **(I)**, aboveground N uptake at the R5 stage in 2020; **(J)**, aboveground N uptake at the R8 stage in 2020.

**Figure 8:**
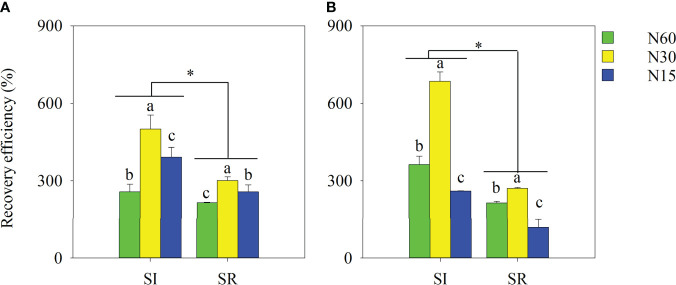
Effects of straw incorporation and nitrogen application on N use efficiency of intercropped soybean at the full-maturity stage. Different lower-case letters indicate significant differences under different N application rates (*LSD*, *P*< 0.05). Data were shown as mean ± S.D. (n=3). The asterisk “*” indicates significant differences (*P*< 0.05) between straw incorporation and straw removal. SI, straw incorporation; SR, straw removal; N60, convention N (60 kg N ha^-1^); N30, reduced N by 50% (30 kg N ha^-1^); N15, reduced N by 75% (15 kg N ha^-1^). **(A)**, the recovery efficiency of soybean in 2019; **(B)**, the recovery efficiency of soybean in 2020.

Compared with SR, the aboveground N uptake per plant in SI was considerably increased during the growing season (**Figure 7**). On average, the aboveground N uptake in SI was notably greater by 34.1%, 54.8%, 33.2%, and 32.5%, respectively, than in SR under the N60, N30, N15, and N0 treatments (**Figure7E-J**). Although the aboveground N uptake decreased with the decrease of N in SI and SR, it was insignificant between N60 and N30 in IS. In contrast, the aboveground N uptake was markedly reduced when the N input was lower than 15 kg N ha^-1^ compared with the N input greater than 30 kg N ha^-1^. Similarly, straw incorporation and N application significantly influenced RE (**Figure 8**). Compared with SR, the average RE in SI significantly increased by 44.3%, 109.2%, and 85.7% under the N60, N30, and N15, respectively. The RE in N30 was markedly greater than in N60 under both SI and SR. The RE in N15 remarkably increased in 2019 and significantly decreased in 2020 compared with N60.

### Carbon dioxide emission

**Figure 9:**
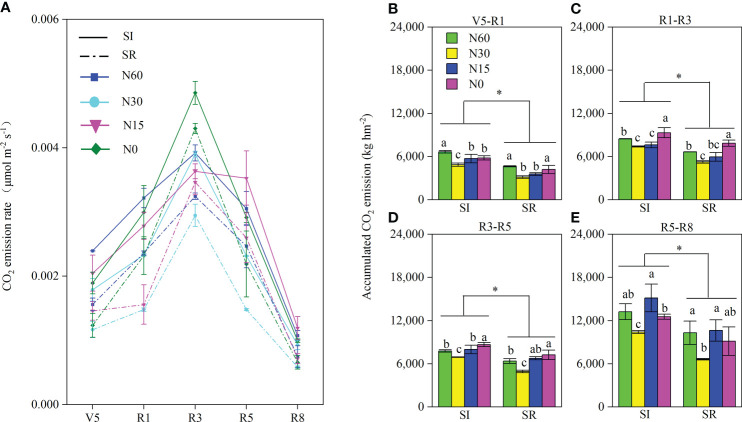
Effects of straw incorporation and nitrogen application on soil CO2 emissions properties. Different lower-case letters indicate significant differences under different N application rates (*LSD*, *P*< 0.05). Data were shown as mean ± S.D. (n=3). SI, straw incorporation; SR, straw removal; N60, convention N (60 kg N ha^-1^); N30, reduced N by 50% (30 kg N ha^-1^); N15, reduced N by 75% (15 kg N ha^-1^); N0, zero N (0 kg N ha^-1^); V5, the fifth trifoliolate stage of soybean; R1, the beginning flowering stage of soybean; R3, the beginning pod stage of soybean; R5, the beginning seed stage of soybean; R8, the full-maturity stage of soybean; V5-R1, the growth stage from V5 to R1; R1-R3, the growth stage from R1 to R3; R3-R5, the growth stage from R3 to R5; R5-R8, the growth stage from R5 to R8. The asterisk "*" indicate significant differences (P < 0.05) between straw incorporation and straw removal. **(A)**, the CO2 emission rate at the different growth stages of soybean; **(B)**, the accumulated CO2 emissions from V5 to R1; **(C)**, the accumulated CO2 emission from R1 to R3; **(D)**, the accumulated CO2 emission from R3 to R5; **(E)**, the accumulated CO2 emission from R5 to R8.

Independent with N input, the soil CO_2_ emission rate was more remarkable in SI than in SR during the cropping season (**Figure 9A**). With the decrease of N input, the lowest soil CO_2_ emission rate was observed in N30 under both SI and SR during the cropping season. The soil CO_2_ emission rate increased from the V5 stage to the R3 stage and decreased from the R3 stage to the R5 stage (**Figure 9A**). Similarly, the accumulated CO_2_ emission was significantly higher in SI than in SR during the cropping season (**Figures 9B–E**). Compared with SR, the accumulated CO_2_ emission in SI was notably increased by 43.2%~60.0% from the V5 to R1 stages (**Figure 9B**), by 18.6%~40.8% from the R1 to R3 stages (**Figure 9C**), by 8.1%~40.4% from the R3 to R5 stages (**Figure 9D**), by 28.2%~57.1% from the R5 to R8 stages (**Figure 9E**), respectively. The lowest accumulated CO_2_ emission was observed in N30 under both SI and SR treatments (**Figures 9B-E**).

## Discussion

Numerous studies have reported that straw incorporation and N application can enhance crops’ N uptake and use efficiency (Baker and Blamey, 1985; Malhi et al., 2011; Xia et al., 2018; Zheng et al., 2021). However, there are also debates. Shan et al. (2012) found that independent of N application rates, the aboveground N uptake and N recovery rates of winter wheat are significantly decreased by straw incorporation in contrast to straw removal. Malunga et al. (2017) reported that the N uptake of intercropped maize is increased by N addition, but the N use efficiency decreased with increasing N input in the maize-legume intercropping. In our present study, the N uptake and recovery efficiency (RE) of soybean in SI was significantly increased in contrast to SR. There was no significant differences in N uptake between N60 and N30 under the straw incorporation treatment. However, it was significantly decreased in N30 in contrast to N60 under the SR treatment. In addition, we found that the averaged RE was significantly increased by 79.7% under the SI treatment in comparison to SR treatment. Compared with N60, the RE in N30 was significantly increased under both SI and SR treatments. However, the RE in N15 was significantly increased in 2019 and significantly decreased in 2020, in contrast to N60. This indicated that the reduced application of excessive N fertilizer is not conducive to the N uptake and utilization of soybean in the wheat-maize-soybean relay strip intercropping system. But, straw incorporation can alleviate the nutrient absorption and yield loss caused by no N application or excessive N reduction. The following aspects regulated the aboveground N uptake increase and intercropped soybean utilization efficiency.

Firstly, the aboveground N uptake increase and intercropped soybean utilization results from the rise of N sources in the soil. In our present study, the soil total N content was significantly higher in SI than in SR under different N application rates during the cropping season. Moreover, soil total N content of SI was insignificant between N60 and N30, but that of SR was remarkably decreased in N30 in contrast to N60. On the one hand, straw incorporation and reduced N application accelerated straw decomposition and promoted the release of nutrients from crop straw by increasing the activity of soil bacteria and fungi (Wang and Bakken, 1997; Moran et al., 2005). On the other hand, the increased soil N content is due to the increased soil urease activity (Zheng et al., 2022). Compared with SR, the soil urease activity was significantly increased in SI. Besides, the highest soil urease activity in SI was obtained in N60 and N30. The increase in urease activity promoted urea hydrolysis and N release.

Secondly, intercropped soybean’s aboveground N uptake and utilization efficiency are related to root growth and absorption capacity (Zheng et al., 2021; Zheng et al., 2022). Root growth affects crop growth, nutrients, and water uptake, and a well-developed fine root system accounts for N uptake and utilization (Zheng et al., 2021). On average, the root biomass of intercropped soybean was significantly increased by 21.8% in SI compared with the SR. This finding is consistent with previous finding**s** (Xu et al., 2018). Crops can increase soil nutrient uptake *via* root proliferation in nutrient-enriched regions (Chilundo et al., 2017). In our present study, the root length, surface area, and volume of intercropped soybean were significantly greater in SI than in SR. The changed root configuration enhanced the root absorption range of soybean in SI treatment. The optimized root configuration help to efficiently use the potential soil resource, and a higher root surface denotes the high efficiency of acquiring (Zheng et al., 2022). Thi**s** i**s** probably the reason why N is efficiently used in SI. Xu et al. (2018) found that straw incorporation coupled with low N input can promote root growth and deep root. In our study, the root growth parameters of intercropped soybean, e.g., root length and surface area, were greater in N30 than in N60 under the SI. In contrast, the root length and surface area of SR significantly decreased in N30 compared to N60. The improved root growth and configuration may be due to enhanced soil physical properties. Xu et al. (2018) found that straw incorporation coupled with low N application promoted root proliferation and growth in deep soil by decreasing soil bulk density. In our present study, SI significantly increased the soil macroaggregates’ stability and porosity, but significantly reduced soil bulk density compared with the SR. This increases the soil permeability and the ability of water and fertilizer conservation, which is beneficial to the root growth of intercropped soybean. A well-developed root growth and distribution can promote root uptake capacity (Zheng et al., 2021; Zheng et al., 2022). The root bleeding sap intensity is an important indicator of root activity, and the components of bleeding sap reflect the nutrients of root absorption and transport (Zheng et al., 2021). In our study, we found that the root bleeding intensity of soybean in SI was notably increased compared with SR. Then, the ammonium-N and nitrate-N contents of bleeding sap were significantly increased in SI at the R3 and R5 stages. The ammonium-N and nitrate-N contents of soybean bleeding sap were greater in SI when combined with N input, especially N input exceeding 30 kg N ha^-1^.

In addition, the increase in aboveground N uptake and utilization efficiency of intercropped soybean is related to soybean nodulation and N fixation (Zheng et al., 2022). Almost half of the N demand in the lifespan for legume growth can be met by biological N2 fixation (BNF) (Salvagiotti et al., 2008). The BNF of legumes not only meets its own growth needs but also provides additional N sources for soil. The BNF of legumes can be inhibited by N application (Hu et al., 2016). The nodulation and N fixation of soybean can be promoted by reducing N input in contrast to conventional N input (Du et al., 2020). With the increase of N input, although the nitrogenase activity and nitrogen fixation potential of soybean nodules were remarkably decreased at the R3 stage, those peaked in N30 at the R5 stage (**Figures 6A-C**). In contrast, the aboveground N uptake was notably increased with the increase of N input (**Figure 7**). In maize-soybean intercropping systems, increasing N input will promote maize growth, then strengthen the interspecific competitive use of resources (Zheng et al., 2021). Thus legumes acquire more N from the soil than symbiosis nitrogen fixation due to resource limitation because symbiosis nitrogen fixation is an extremely energy-consuming process (Tjepkema and Winship, 1980). Moreover, we found that the nodule number, nodule dry weight, nodule nitrogenase activity, and nodule N fixation potential of soybean were significantly increased with the decrease of N application rate under both SI and SR treatments. Compared with SR, the nodulation and N fixation capacity of soybean were significantly enhanced by SI. Because competitive use of N between microorganisms and crops will decrease soil N when the straw is incorporated (Wang and Bakken, 1997). Then, the decrease of N content in soybean rhizosphere soil promotes soybean nodulation. Furthermore, Siczek A (2011) indicated that soybean’s nodulation and N fixation ability could be enhanced by reducing soil compactness. Although straw incorporation significantly reduced soil bulk density, the soil macroaggregates stability and porosity notably increased. Finally, it was beneficial to nodulation and N fixation of soybean.

Straw incorporation not only increased N uptake and crop yield but also increased soil carbon emissions (Bhattacharyya et al., 2012; Zhao et al., 2019). In this study, straw incorporation significantly increased soil CO_2_ emission rates and accumulated CO_2_ emissions in contrast to straw removal under different N application rates. With the advance of the soybean growth process, the soil CO_2_ emission rate increased at first and then decreased, and the highest CO_2_ emission rate was observed in the R3-stage. Straw incorporation promoted soil microorganisms’ activity by increasing soil C and N sources and improving soil physical properties (Li et al., 2018). In addition, the increase in CO_2_ emission is related to the growth of the root system. This is probably due to the root exudates promoting microbial respiration (Badri and Vivanco, 2009). A previous study indicated that straw incorporation coupled with high N input could increase CO_2_ flux (Pan et al., 2006). On the contrary, Zhang et al. (2020) found that straw incorporation coupled with reduced N input significantly decreased CO_2_ emission in contrast to conventional N. Zhou et al. (2021) indicated that reduced N application combined with long-term reduce/zero tillage could significantly decrease soil C emissions. In this study, the soil CO_2_ emission rates and accumulated CO_2_ emissions significantly decreased in N30 compared to N60 under the SI treatment.

## Conclusions

Our results indicated that the soil GMD of macroaggregates, soil total porosity, soil total N content, and soil urease activity were greater in SI than in SR. However, the soil bulk density in SI significantly decreased. Compared with SR, the root length, root surface area, volume, and biomass of soybean in SI were notably significantly increased. The root length and surface area were greater in N30 than in N60. Furthermore, soybean nodulation and N fixation in SI was markedly higher than in SR. Compared with convention N input, reduced N input significantly increased nodulation and N fixation of soybean. The aboveground N uptake and RE of soybean in SI significantly increased in contrast to SR. The highest RE of soybean was observed in SI with N30 treatment. Besides, the soil CO_2_ emission rate and accumulated CO_2_ emissions were significantly higher in SI than in SR. But, those were considerably decreased in N30 in contrast to N60. In conclusion, the increased N uptake and utilization were due to the improved soil properties, root N uptake capacity, and enhanced nodulation and N fixation of soybean. Straw incorporation coupled with 30 kg N ha^-1^ input was a sustainable strategy in the wheat-maize-soybean relay strip intercropping system. It significantly increases N uptake and utilization of soybean and reduces soil CO_2_ emissions.

## Data availability statement

The original contributions presented in the study are included in the article/**Supplementary Material**. Further inquiries can be directed to the corresponding author.

## Author contributions

BZ, PC, and TY conceived and designed the experiment. BZ and PC performed the statistical analyses. BZ, PC, QD, HY, and KL were involved in field data collection. All authors contributed to writing the paper. All authors contributed to the article and approved the submitted version.

## Funding

The work was supported by the Grant from the National Key Research and Development Program of China (2021YFF1000500), the Program on Industrial Technology System of National Soybean (CARS-04-PS18), and the National Natural Science Foundation of China (31872856).

## Conflict of interest

The authors declare that the research was conducted in the absence of any commercial or financial relationships that could be construed as a potential conflict of interest.

## Publisher’s note

All claims expressed in this article are solely those of the authors and do not necessarily represent those of their affiliated organizations, or those of the publisher, the editors and the reviewers. Any product that may be evaluated in this article, or claim that may be made by its manufacturer, is not guaranteed or endorsed by the publisher.
