# Supplementary material for: Straw incorporation and nitrogen reduction effect on the uptake and use efficiency of nitrogen as well as soil CO2 emission of relay strip intercropped soybean
Source: Front Plant Sci. 2022 Nov 9;13:1036170. doi: 10.3389/fpls.2022.1036170 (PMC9928161; doi:10.3389/fpls.2022.1036170)
Supplement: Supplementary file 1 [file DataSheet_1.docx]

**Supplementary files**

Figures captions

Fig. S1 Precipitation and temperature during the soybean cropping seasons.

Fig. S2 Schematic diagram of planting pattern.


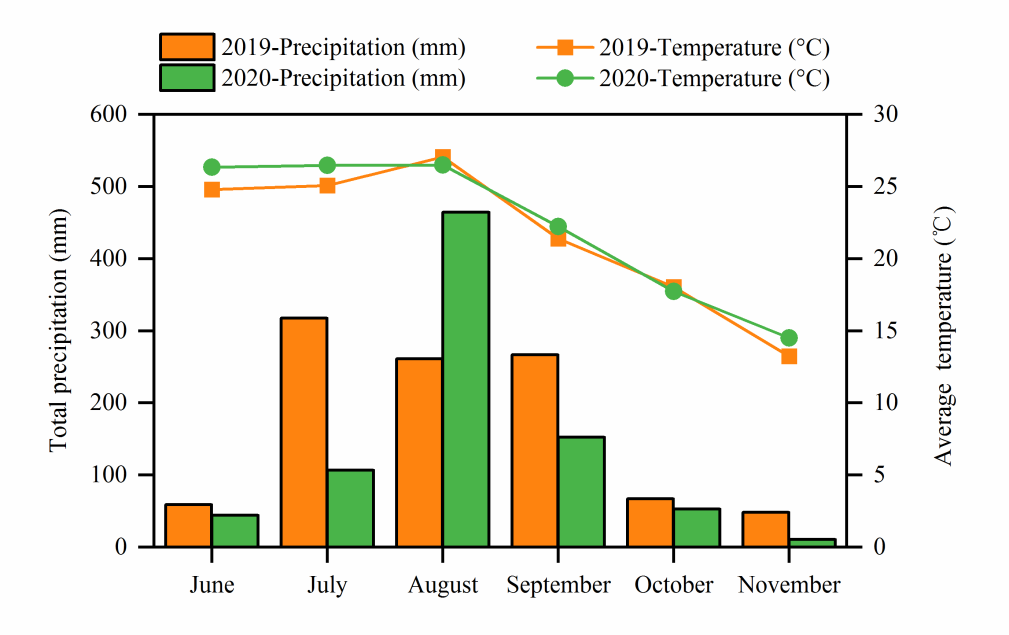


Fig. S1 Precipitation and temperature during the soybean cropping seasons.


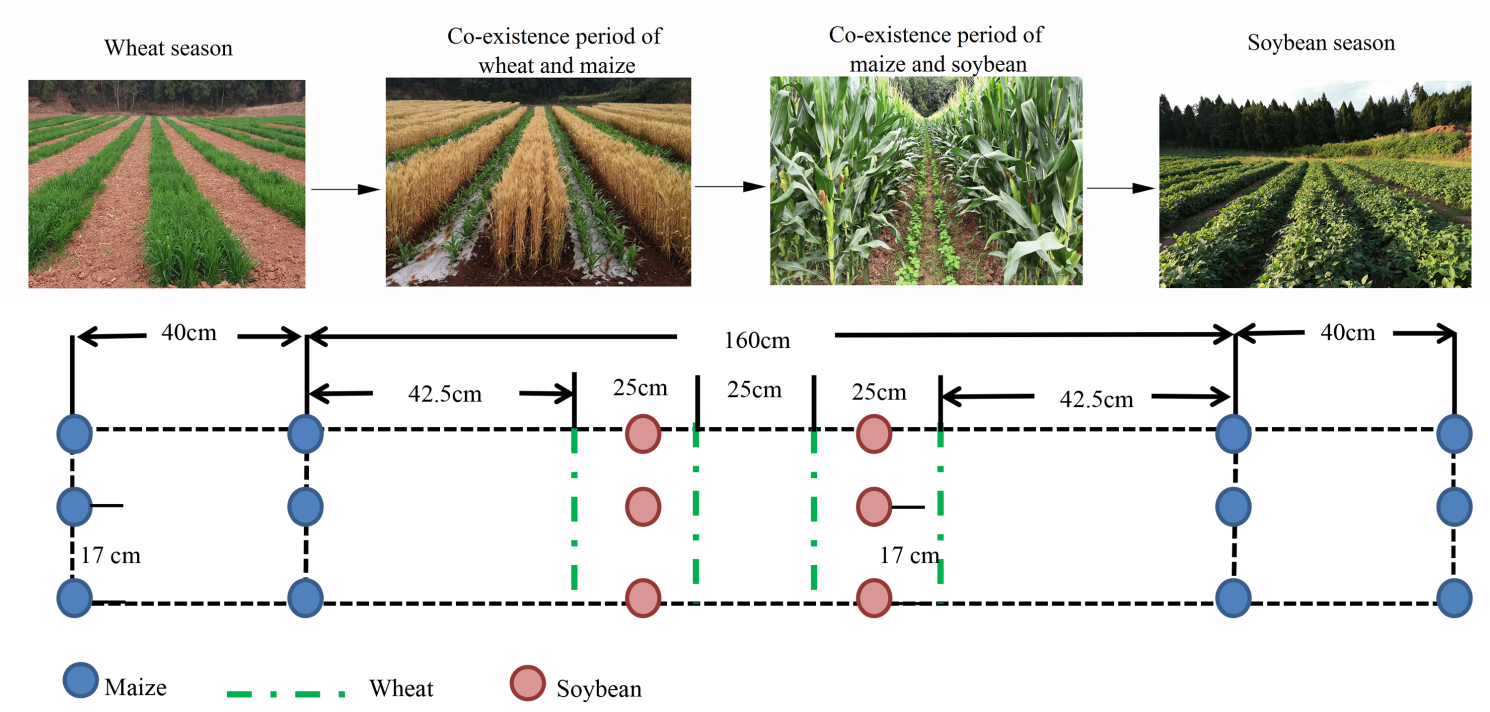


Fig. S2 Schematic diagram of the maize-soybean-wheat relay strip intercropping.
